# “To be healthy to me is to be free”: how discourses of freedom are used to construct healthiness among young South African adults

**DOI:** 10.1080/17482631.2019.1603518

**Published:** 2019-04-29

**Authors:** Michelle De Jong, Anthony Collins, Simóne Plüg

**Affiliations:** a School of Public Health, Faculty of Community and Health Sciences, University of the Western Cape, Bellville, South Africa; b Faculty of Arts and Design, Durban University of Technology, Durban, South Africa; c Department of Social Inquiry, La Trobe University, Melbourne, Australia; d Department of Psychology, Rhodes University, Makhanda, South Africa; e International Centre for Non-Violence, Durban University of Technology, Durban, South Africa

**Keywords:** Freedom, health, neoliberalism, work, social constructionism, discourse analysis

## Abstract

**Purpose:** Healthiness is constructed, in Western culture, as a moral ideal or *supervalue*. This paper will interrogate the assumption that health and the pursuit of healthiness is always and unquestionably positive, by exploring how discourses of health and freedom interact to reinforce the current inequalities and detract from social transformation. **Method:** Twenty young South African adults were interviewed about their understandings and experiences of health. These discussions were analysed using Foucauldian discourse analysis. **Results:** Participants constructed healthiness as facilitating the experience of freedom, while at the same time being dependent on a personal orientation towards freedom (as opposed to merely submitting to dominant health authorities). Freedom discourses also played a role in connecting health to neoliberal discourses idealizing economic productivity and hard work. Participants were able to construct a self that is active, productive, valuable, hopeful, and self-assured when talking about health using discourses of freedom. However, these discourses also functioned to moralise and idealise healthiness, which contributed to blaming poor health on its sufferers. **Conclusion:** Health/freedom discourses can further reinforce the neoliberal value of individual responsibility by constructing self-improvement and self-work as the solution to ill-health, thereby contributing to victim-blaming and weakening support for public health interventions.

Freedom is also unique in that it is the mother of all values. If we consider such values as honesty, love, or courage, we find, strangely enough, that they cannot be placed parallel to the value of freedom.

May, 1981, p. 6

“There is more than one kind of freedom,” said Aunt Lydia. “Freedom to and freedom from. In the days of anarchy, it was freedom to. Now you are being given freedom from. Don’t underrate it.”

Atwood, 1986, p. 34

“The truth is that we are not yet free; we have merely achieved the freedom to be free, the right not to be oppressed. We have not taken the final step of our journey, but the first step on a longer and even more difficult road. For to be free is not merely to cast off one’s chains, but to live in a way that respects and enhances the freedom of others. The true test of our devotion to freedom is just beginning.”

Nelson Mandela

## Introduction

Foucault (2008) emphasizes the importance of critiquing “institutions that appear both neutral and independent” (p. 41). Health is a concept which is generally identified as being an ideal to aspire towards and an objectively good state to pursue. This paper will argue against the assumption that the pursuit of healthiness is always and unquestionably positive by examining the way discourses constructing healthiness, in this specific case discourses of freedom, can function to reinforce current social inequalities and preclude positive social transformation. In particular, these discourses will be situated within the context of neoliberal capitalism (which idealizes individual responsibility and productivity) and healthism (which moralizes healthiness). This critique of popular health discourses is not intended to diminish the life and death consequences associated with a lack of access to healthcare and preventative resources but instead to suggest that some of the current ways in which health is constructed and talked about actively impede the changes necessary to ensure that health and health care resources are easily accessible to everyone.

## Background and literature review

This section will briefly outline the relevance of neoliberalism in relation to discourses of freedom and health. The concept of freedom will then be explored in more depth, focusing on its value in contemporary societies, including South Africa and how it relates to health. Finally, the concept of healthism will be addressed, introducing one of the dominant health discourses of relevance to the present discussion.

This research is located within ongoing efforts to reduce inequality and promote well-being in South Africa, however with respect to health, it diverges from both the state project of implementing public medical services, and the tacit project of globalised lifestyle media and consumer culture linking health to the marketing of personal lifestyle choices. This latter popular cultural form is in fact the object of investigation in this study. Our assumption is that it is only by formally identifying this network of ideas and critically examining how it functions, both in constructing individual identities and legitimating social policies, that we can identify some of the negative consequences of this specific construction of “healthiness”.

### Neoliberalism

Neoliberalism is the most recent form capitalism has taken, and is characterised by deregulation of market forces and the reduction of social welfare programs, amongst other social, political and global changes (Kotz, 2015). Although neoliberal economies tend to be characterized by cuts in welfare spending in the USA and European countries, South African health care spending has been increasing year on year (2010–2015 South African National Treasury, cited in Moult & Müller, 2017). However, in more recent years health care spending as a percentage of government spending has plateaued (UNICEF, 2017). In addition, because of the high levels of poverty and inequality in South Africa many of the health care problems associated with neoliberal austerity measures are also faced here (Moult & Müller, 2017). These include issues like an under-resourced public health system, the increased reliance on privatized health care, and difficulties in access public health services (Quaglio, Karapiperis, Van Woensel, Arnold, & McDaid, 2013). In addition, “neoliberalism, as an ideology that holds market exchange and economic rationalism as ethics in themselves, and as being capable of acting as a guide for all human action, has seeped into public service provision in South Africa as much as elsewhere internationally” (Moult & Müller, 2017, p. 219; Harvey, 2005). In this paper, the focus will primarily be on the, “general style of thought, analysis and imagination,” (Foucault, 2008, p. 219) that the globalisation of neoliberal economies has fostered. The social values that characterise neoliberalism include individualism, competition, and personal freedom. The notion of freedom is especially significant for the focus of this paper and will be discussed more below.

### Freedom

Freedom can be understood as the power people have to help themselves, to have an effect on the world, and to determine action without being hindered. As an ideal it is highly valued in many contemporary societies. Varman and Vikas (2007) regard freedom as, “one of the most celebrated of the human values,” (p. 117). Some even consider freedom to be, “central to human existence,” (Varman & Vikas, 2007, p. 118; Sen, 2000). In South Africa, in particular, freedom has a deep significance because of the massive violations of freedom that occurred during Apartheid political era. Oliver Tambo, a leading anti-apartheid activist, expressed the particular importance of freedom in South Africa when he emphasised that “The fight for freedom must go on until it is won; until our country is free and happy and peaceful as part of the community of man, we cannot rest” (Tambo, 1967).

Freedom is often articulated as either freedom *from* or freedom *to*. To be considered free one must be liberated *from*, “bondage or slavery,” (Rose, 1998, p. 62) and to be empowered *to*, “do as one likes” (Rose, 1998, p. 62). This paper will focus more on discourses of freedom which relate to a freedom *to*. Bauman (2000) argues that feeling free depends on a balance being achieved between “the wishes, the imagination and the ability to act: one feels free in so far as the imagination is not greater than one’s actual desires, while neither of the two reaches beyond the ability to act.” (p. 17). Freedom is also closely linked to the idea of empowerment. In order to be free, individuals need to be empowered so that they can exert their will, and so that they have the ability to act. Varman and Vikas (2007) describe empowerment as the “enhancement of social, political and economic strengths of an individual so that s/he can resist domination of any form.” (p. 118). Empowerment and health are often linked together, and empowerment is often an explicit goal of health promotion efforts (Grace, 1991; Laverack, 2009; Rissel, 1994).

Empowerment and freedom are also deployed in discourses around competitive capitalism. In Milton Friedman’s (1962) book Freedom and Capitalism, he claims “I know of no example in time or place of a society that has been marked by a large measure of political freedom, and that has not also used something comparable to a free market to organize the bulk of economic activity” (p. 16). Rose (1998) argues that it has been neoliberal thinkers who have been one of the most vocal and most powerful advocates of individual freedom over the thirty years before the end of the 20th Century.

However, Davies (2015) puts forth the argument that the competitive culture fostered by the current form of capitalism can have a number of detrimental health effects including depression, anxiety, and character traits such as obsessive perfectionism, which can manifest in unhealthy behaviours. So, although capitalism is purported to facilitate freedom, the kind of competitive culture that accompanies this economic system can impact negatively on health, although health itself is viewed by the participants as key to the experience of freedom and empowerment.

Freedom has come to be the ideal used to legitimate our political systems- “the free world” as opposed to dictatorships; our economies- the “free market” as opposed to centrally planned economies and even our understanding of the self; and what it means to exist as a person- the “freedom-loving authentic individual of now” as opposed to merely one of a collective (Rose, 1998). Freedom provides us with a personal ideal to aspire to, we are encouraged to pursue freedom for ourselves, as opposed to collectively, through practices of self-improvement and through the way in which we organise our lives. Freedom is valued so highly that some view it as a legitimate reason to wage wars, exert domination and commit atrocities all over the world. There seems to be little disagreement over the idea that humans should be free, and that the way society is organised, and the way that we engage with ourselves and others, should reflect this (Rose, 1998).

Bauman (2000; Verhaeghe, 2012, p. 57) comments on the strange contradiction we experience in the context of modern, capitalist societies: “Never have we been so free. Never have we felt so powerless”. Verhaeghe (2012) argues that this is because, although individuals (specifically the privileged middle-class) have so many more options than at other points in history, their choices have no broad political consequences, they are insignificant. Whether people choose to go for a run or do aerial yoga, makes no material difference in relation to society more broadly or to anything beyond themselves. He also points out that in order to have access to the vast freedom and individual choice that individuals are promised through consumer capitalism, they need to be successful in accordance with the narrowly defined terms that will support our economic system- they need to make money. Due to very high levels of poverty and inequality, especially in South Africa, this is not accessible for most people. The discourses discussed in this paper construct freedom as dependent on health and, as a result, exclude individuals who are dealing with issues relating to poor health from experiencing freedom. These discourses of freedom and health also function to reproduce the status quo, as underlying assumptions about the ability of individuals to *choose* health and therefore to liberate themselves from physical restrictions, serve to construct those who are oppressed by the physical and social consequences of poor health as responsible for their own situations. As a result, this vilifies the “unhealthy”, and sanctions a societal abdication from responsibility to work towards social support for vulnerable and disadvantaged people. This kind of moralisation around health has developed in the context of the increasing prominence of healthism discourses, described below.

### Healthism

In 1980, Crawford coined the term “healthism” which refers to the increasing moralization of health that has occurred. Crawford (2006) argues that this moralizing is cultivated by health promotion efforts which have led to the increasing medicalization of our lives. It has been argued that health, which was previously confined to the area of biology and disease, has permeated into almost all aspects of life (Metzl, 2010). Health then starts to become viewed as a “moral imperative” (p. 6) and an essential aspect of social status and self-worth (Metzl, 2010). Petersen (2015) argues that healthism, “promises salvation of the self- resurrection or transcendence through intensive work on the self” (p. 7). In other words, health, specifically through intensive self-governance, has become closely tied to moral redemption. Halse (2009) discusses the pressure to adopt healthy habits as a component of being a responsible “bio-citizen”. The implication here is that individuals are expected to take responsibility for ensuring their own good health as the effective operation of a society depends on it. The importance placed on health and its idealisation was clearly visible in this study, specifically through the linking of health to discourses of freedom.

A number of researchers have explored how health is constructed within different populations. Many of these studies support Crawford’s (1980) observation of the pervasiveness of healthist discourses in understandings, experiences and discussions around health and what it means to be a healthy citizen (Gard & Wright, 2001; Wright & Burrows, 2004). These studies also explore additional, more specific discourses (which may fall under the umbrella of healthism) which have also been found to be significant when constructing meanings and understandings of health. For example, Wright, Flynn and MacDonald (2006) discuss the notion of health as “enabling” specifically in relation to young men’s constructions of fitness. The participants talked about how a certain level of fitness enabled them to participate, to enjoy, to compete and to perform. Numerous studies (mostly from western contexts) have documented the construction of health as closely associated with, or even equivalent, to a certain physical appearance which is usually defined in accordance with western media ideals of attractiveness (Kirk & Colquhoun, 1989; Norman, 2011; Wright, Flynn & MacDonald, 2006). Although very little research directly explores how South African individuals construct health, Barnes and Milovanovic (2015) note an increasing prevalence of “behaviour change discourse” (which frames health as the result of individual lifestyle choices) in the South African context. This provides a useful example which illustrates how cultural globalisation has led to the increasing permeation of neoliberal ideology.

## Aim

Drawing on the scholarly literature in this developing field, this study aims to map how understandings of healthy lifestyles are constructed, and critically engages with the assumption that the pursuit of health and wellness is always necessarily positive and will inevitably lead to a better, more fulfilling life. It also explores how consumer capitalism provides the context for certain health discourses to become ascendant, and how these discourses support and reproduce those very social arrangements. More specifically, this paper aims to provide a detailed analysis of the ways in which participants used discourses of freedom to construct health, while at the same time reinforcing the prominence and moralisation of health in our society as well as legitimating discourses of productivity and work.

## Methodology

The study which this article is based on investigates how ideas of health are constructed by young adult South Africans. It is a qualitative study which draws on a social constructionist theoretical framework.

### Sample

Purposive and snowball sampling were used to recruit 20 young adult South Africans (10 men and 10 women) living in urban areas of Durban, Cape Town and Johannesburg (Please see Table I for more information about the demographics of participants). Individuals able to provide a range of perspectives on health were approached. This included, for example, individuals who were interested in health but are faced with the economic challenge of accessing health improvement resources, students studying to become medical practitioners, individuals interested in “alternative” health practices such as eastern medicine and yoga, individuals interested in “lifestyle blogging” and those who described themselves as not consistently engaging in health-promoting behaviours. These individuals were accessed through the authors’ personal and professional networks and through local fitness centres and yoga studios in order to gain access to individuals who worked in health and fitness environments. From there, snowball sampling was used to gain access to additional participants in order to increase the pool of interviewees. Although effort was made to recruit participants from diverse backgrounds, including varied social classes and historically defined racial categories, the majority of the participants were from middle-class backgrounds and many (13) identified as “white”. This sample is not intended to be representative of any subsection of the South African population and details on participants are provided below for the purposes of contextual specificity and not for the purposes of generalisation.

**Table I.** Sample description.

Pseudonym	Age	Race (As described by participants)	Gender	Occupation
Adele	40	White	Female	Teacher
Alex	23	White	Male	Unemployed
Amelia	18	Black	Female	Student
April	26	White	Female	Dance Instructor/ Health Blogger
Ben	26	White	Male	Yoga Instructor/actor
Callie	20	White	Female	Student
Christina	23	White	Female	Student
Derek	21	White	Male	Student
George	22	Black	Male	Student
Isobel	26	Indian	Female	Doctor
Jackson	25	Coloured	Male	Fitness Manager
Jo	22	White	Female	Unemployed
Lexi	28	White	Female	Teacher’s Assistant
Mark	25	White	Male	Student
Meredith	21	White	Female	Student
Miranda	24	White	Female	Sales Agent
Nathan	35	Coloured	Male	Student
Owen	37	White	Male	Equity Salesman
Preston	22	Indian	Male	Student
Richard	19	Black	Male	Student

### Data collection

In depth semi-structured individual interviews were used to collect data from each participant. Interviews took place in locations chosen by the participants and ranged from coffee shops and fitness centres, to the participants’ and the interviewer’s homes. Some of the question that participants were asked during these interviews included: What does health mean to you? What do you think makes a person healthy or unhealthy? Would you say that you are healthy? Why? Do you think it is important to be healthy? Why? All of the interviews were transcribed, verbatim, and transcripts included details of other vocalisations indicating emotion or opinion, such as laughter, expressions of disgust and of disapproval.

### Data analysis

Data was analysed using Foucauldian discourse analysis. Parker (1994) defines discourses as, “sets of statements that construct objects and an array of subject positions,” (p. 245). A discourse is made up of certain assumptions that are often taken for granted as true (Cheek, 2004). Within this approach, our identities, social relations and the way we experience our environment are all viewed as constructed through the language we use at specific moments in history (Burr, 1995; Cruikshank, 1999; Scott, 1992). In this study, Willig’s (2013) version of discourse analysis was used. This method follows 6 steps which involve carefully examining the language used by participants, unpacking how discourses structure the participants’ speech, and exploring how these discourses function to construct both the discursive object which is being discussed (in this case, health) as well as the subject who is speaking. Willig’s (2013) analysis method also pays specific attention to exploring the feelings, experiences, and behaviours which are facilitated by different discourses, as well as how discourses connect to, and differentiate from, each other.

### Ethical considerations

All participants were given informed consent forms which were explained to them. They were made aware of the purpose of the study, what information was going to be used, how much time it would require should they choose to participate, and how the information they provide would be stored and disposed of. The participants were also informed about who would have access to the transcriptions of the interviews. Consent to audio-record all interviews was obtained. Participants’ personal details were kept confidential and their names, as well as the names of others they refer to, were changed to uphold anonymity. It was made clear that participants were free to withdraw from the study at any stage up to publication.

## Results

Discourses of freedom are used to structure a number of the participants’ speech about health, constructing health, freedom and participants’ identities in specific ways. Their use of these discourses in our conversations will be explored along with the functions that they perform, the social, political and economic structures they are facilitated by and uphold, as well as some of the consequences they may have.

### Health as freeing

Health is constructed by many participants as facilitating the experience of freedom. In fact, in some cases healthiness and freedom are constructed as synonymous. Discourses of freedom are employed by a number of the participants in this study when describing what health is and why they believe health is so important (they all said that it was).

Jo: To be healthy, to me, is to be free…There’s a reward for your effort. And that’s what it feels like for me, it’s something freeing.

April: I think it’s being balanced, actually being able to also live your life in a… I don’t know what the word is… freely! Completely free of you know like pressure, you know from yourself, I think that’s healthy.

Freedom discourses when used in discussions around health often result in the understanding of health as an avenue to the experience of freedom.

### Health, freedom and opportunity

Many of the participants discuss the idea that being healthy allows for opportunities. Health was framed as empowering individuals to choose what they wanted to do without constraint. In the following quotes, participants use vague phrases such as ‘*allow you then to operate optimally‘* and *‘to do what you need to do‘*. There is a general lack of specificity in the particular activities they would like to be doing and that a state of healthiness allows for. This indicates that the specific activity is not really the point and instead, it is the opportunity to choose what one will do without restriction that is appealing. Being healthy is understood to allow them a sense of freedom in how they live and what they can choose to do. Health is constructed as facilitating opportunities for action and the freedom to function “normally”.

Christina: Ja and its [health] just all the different aspects that make you a sort of functioning human on a day to day basis.

Isobel: …also being able to function well and kind of play a role in society as well.

Owen: They have better energy, they have better capacity, and they have a socially positive approach to life…

Owen: …all sorts of routines that support your health that allow you then to operate optimally.

Christina: I think also we construct sort of roles and plans for our lives and dreams for our families and a lot of that is reliant on our health and to fulfil those roles

Health is also understood as a tool to overcome obstacles and as a source of protection against potential limitations. Phrases like, “*live your life … freely”*, “*make you a sort of functioning human on a day to day basis”, “function well”, “better capacity”, “all sorts of routines”, “do what I need to do”* show how the things that health enables participants to do are wide-ranging. From this point of view, it appears that health is necessary for any number of activities or goals. This is related to Wright, Flynn and MacDonald’s (2006) findings where fitness was constructed as “enabling”. It is also a possibility that it is not only that their health needs to be good in order to complete certain tasks or achieve certain goals but also that good health *facilitates* the life they *want*. Bauman (2000) provides a possible explanation as to why improved health is considered key to the experience of freedom when he argues that in order to achieve a balance between our desires, our imagination, and our ability to act, we can either moderate our desires and imagination or we can improve our ability to act. It may be that these participants are attempting to improve their ability to act through the improvement of their health. It is possible that in a culture which professes that anything is possible, our desires and imaginations have soared, whereas our ability to act is restricted and health improvement strategies provide us with an opportunity to attempt to bring the three into balance. Importantly, within these quotes, this synchronisation of desires, imagination and ability to act requires the individual to adapt in order to function successfully within societal constraints. Society is not required to change to better facilitate individual action, rather the individual makes themself free by optimising their health through lifestyle choices

Owen’s phrase that associates health with practices that, “*allow you then to operate optimally”* may be understood using Foucault’s (1978) concept of “anatomo-politics”, which describes the way in which biopower functions at the individual level. He explains that bodies are understood as a machine, and institutions function in ways that discipline individual bodies so that they may be subjugated and managed (Foucault, 1978). Dominant discourses about what it means to be a good citizen may have been internalized and enacted through technologies of the self, specifically health improvement practices. We see here an ambiguity between the construction of health as enabling individuals to choose freely how they want to live, and health as a requirement necessary for individuals to adequately fulfil their social roles.

### The freedom to choose your healthy

Participants also made use of discourses which foregrounded the “free to choose” discourses in relation to the specific health practices that they engaged in. Participants resisted discourses of healthy behaviours as restrictive or unpleasant and emphasised the importance of being free to choose how they did their health (Pelters, 2014). But in order for health not to be restrictive, there cannot be specific criteria for healthy people and healthy bodies. A number of participants mentioned how it is unclear whether someone is healthy or not, and health often depends on individual situations and is a very personal issue.

Jo: …*people are different, like my body type is different from your body type, my diet is different from your diet. So it needs to be something that can suit you as a person. I think it needs to be something very personal.*

From the responses the participants gave, it was implied that there are many different personal ways to “do health”. Making use of this discourse serves the speaker in that it allows them to avoid being seen as judgmental, and it helps them to avoid guilt or shame if their behaviours or lifestyles do not conform to the expectations related to ideal healthiness. It also allows for the experience of self-determination, sovereignty and empowerment. This is linked to the idea put forth in *The Happiness Industry* that in modern societies there is a lot less authority and a lot more relativism in how we define what is good and what is bad than at previous points in history (Davies, 2015). This discourse is appealing to users as, in theory, individuals can construct a lifestyle for themselves and choose freely what activities they would like to engage in and feel no obligation to participate in activities or conform to rules that they find undesirable. The flexibility in interpretations of what constituted a healthy lifestyle and a healthy person is also essential in order to produce a self which can be secure and accepted, and which is not overwhelmed by guilt and anxiety as a result of a single, rigid set of standards which may be impossible to live up to. However, even though participants make use of this discourse of health as being a practice which is customizable and not prescribed, it is not always experienced as true as a number of activities were identified as clearly unhealthy (smoking, excessive drinking). The assumptions made about what healthy behaviours entailed also tended to contain similarities which reveal that there are specific practices that are generally accepted as healthy- exercising, eating fruits and vegetables and avoiding fried or overly processed foods. These kinds of informally agreed specifications contribute to the stigmatization of those who do not comply with idealised health practices. Metzl (2010) explains that health with its criteria of what is normal and good splits individuals into groups, and those that fall outside of the range of acceptable health indicators- those who are disabled (Lalvani, 2015), ill (Parsons, Bond, & Nixon, 2015) or overweight (Brewis, SturtzSreetharan, & Wutich, 2018)- become victims of stigma.

Discourses of freedom relating to the freedom to choose how to practice health were also noticeable when participants discussed their thoughts on whether or not we have a responsibility towards the health of others. Participants seemed anxious to distance themselves from the idea that individuals should be told what to do, or that their individual freedom should be impinged on in certain ways. Even though all participants said health was essential and should be a priority they did not feel that it was acceptable to inflict this belief onto anyone else.

Lexi: …it’s quite tricky ’cause I don’t want to tell people what to do and how to live their lives, and it’s actually not my responsibility to tell people what to do…

Alex: Maybe like raise concerns about their health but I wouldn’t get directly involved because I mean they their own person, they’ve made their own choices…

April: …I don’t think we have a right to force anybody into changing their lifestyle…

We see here that discourses of freedom are being used in ways which limit the possibility of feeling responsible for the health of others and of viewing the self as intimately connected to others and their health. This discourse creates a distinction between individual health and group health. This may function in ways which diminish the likelihood of collective social action aimed at improving the health of others or the general population, as individuals are responsible for only their own health. It also constructs individuals as the only ones responsible for their illness and the only ones who can overcome it. This focus on personal or individual responsibility can lead to victim-blaming (Ehrenreich, 2009; Goldacre, 2009; Willig, 2011). In this context, victim-blaming refers to the belief that individuals deserve the poor health that they suffer from and that they brought it upon themselves (Ehrenreich, 2009; Goldacre, 2009; Willig, 2011).

There was one notable exception to the view that individuals should be free to choose the health practices that they do or do not engage in, and that specific health practices should not be imposed on anyone.

Jo: I really like the whole- China has that rule where they make everyone do exercises for 30 minutes a day. Like some Chinese businesses actually take time out of the day and they’ll go up on the rooftop to do like Taekwondo or Tai chi or something together which is great. It’s almost like a law to do 30 minutes exercise, in that case, it would be really great- we don’t have the law enforcement to…

This participant also described health as extremely personal and as a freeing experience, however, in this instance she is arguing for health to be overtly enforced. This ambiguity suggests that individuals are free but within limits. They can have as much freedom as they want as long as their choices remain within the boundaries containing behaviours that are likely to improve productivity and prevent illness. This relates to Riley, Thompson and Griffin’s (2010) argument that neoliberalism is inherently contradictory as individuals are meant to experience themselves as having the freedom to make choices that fundamentally shape their lives, while at the same time only being permitted to make fairly specific or “appropriate” choices.

As discussed above, discourses of freedom were used by participants to talk about how health should or shouldn’t be done. In the following section, the way discourses of freedom were used to construct how healthiness was experienced will be explored.

### Health, freedom and energy

The discourse of freedom is also seen when participants use terms like “*lightness*” or “*energetic”* to describe what the state of being healthy *feels like*.

Lexi: … you do feel a bit more revitalised or energetic…

Nathan: …*he has more energy to do things where obviously if you’ve got the weight problem- I mean it can have an impact…*

Isobel: …like in my mind a happy healthy person like gets more done in the day and might be more active…

Owen: It feels well it feels very expansive, it feels very motivating…

Jo: …*it feels like light, not even sunlight but just like the feather light. Like not weighed down but very like free, yes.*

These ideas of “*energy”* being “*revitalised”* and “*feather light”* are considered in Bauman’s (2000) *Liquid Modernity*. When describing the condition of modern society as liquid he explains how we view liquids as light, how lightness leads to mobility and when we travel light we are free to move more easily and quickly. He also discusses how liquids can move past obstacles and are not easily restricted. This captures the sense of liberation that the participants describe as a being result of good health. They describe feeling “*active”, “expansive”* and “*free”*. They can do what they want without being held back or “*weighed down*”. The healthy person is active and adventurous within this discourse. They are capable of doing anything they set their minds to and that is usually something productive and energetic as opposed to something relaxing or passive. We see again in these quotes that the emphasis is having the option to do whatever one pleases and not necessarily a specific activity that requires energy. This idea seems to relate to a kind of fantasy life where all dreams are possible. These are not specific goals that individuals intend to realistically pursue, but rather the idea that there is hope. In the fantasy, there is the option to have more, the future is bright and there is the hope of improvement. This hope might be comforting when confronted with dwindling career options and social and economic problems that cannot be controlled and could negatively impact the ideal future that is hoped for. This notion of hope in relation to health is discussed in Petersen’s (2015) book *Hope in Health*. He notes that in neoliberal societies, hope, specifically the hopes of individuals for themselves, are often attached to “the ‘freedoms’ to pursue certain suggested practices or ‘technologies of the self’” (p. 7).

Discussions of freedom and *lightness* and *energy* are supplemented by an avoidance of heaviness or being sedentary. This idea was both used to subtly criticize bodies labelled “fat” or “lazy”, but also to defend against certain other kinds of ideal healthiness as is seen in Nathan’s quote below. Certain forms of health were evaluated as superior to others based on their facilitation of freedom as mobility and agility.

Nathan: I would never want to do bodybuilding because if your muscles are too big I feel like you’re not mobile, you not agile, so I’m quite a big built person but I prefer being toned because I feel like I can still have a little bit of speed and agility so um I’m feeling I basically want to be more toned…

Nathan explained his preference for a certain kind of fitness as it facilitated action- he could move and he experienced himself as agile when at this level of fitness. This also protects him from pressure to do “*bodybuilding”* which would require intense dedication, commitment, effort and sacrifice as he frames it in a way that makes it restrictive and therefore unappealing- *“you’re not mobile, you not agile”*. This kind of defensiveness is often seen in the participants’ speech in this study. The emphasis in Nathan’s quote on having a body that is functional and effective rather than aesthetically idealised, was also noted in a study by Plüg and Collins (2013). Participants in their study on South African men and body image made similar defensive identity moves to protect themselves from feelings of failure or insecurity and to foster a positive self-concept. In some cases, it seems that attachment to the healthism discourse allows individuals to remove some of the pressure they feel to pursue a specific physical appearance such as one where, *“your muscles are too big”*. Health is seen as superior to attractiveness, and so if individuals can take pride in their bodies as a result of their healthfulness this allows them an avenue of self-acceptance that was possibly not open to them through the pursuit of unattainable beauty ideals. However, in specific ways health is experienced *through* the attainment of a certain appearance, and in some cases health is conflated with beauty ideals. Some participants mention the benefits to their self-esteem that health brings them but this is related to their healthy lifestyle enabling them to lose weight or become “*toned*”. We see evidence of health being co-opted by the fashion and beauty industries, as the appearance of good health is sold as the same thing as the physical experience of health.

Although the participants were typically vague about the specific freedoms made possible by their good health, one that was made explicit was the ability to work. Health was framed as allowing individuals the freedom to work productively.

### Freedom and productivity

The discussion below addresses the notions of freedom and health in relation to productivity and the work environment. Participants emphasised the idea that being healthy was necessary in order to have the option to work. Ill-health restricted productivity while good health ensured that individuals were free to work efficiently and without hindrance. Here, freedom is constructed as the avoidance of any possible limitations to productivity which could result from health problems. The quotes below explicitly mentioned the importance of health in order to allow individuals the opportunity to work.

Isobel: So either you don’t have any disease and you just doing your normal life, you’re having a relationship, you working or whatever…

Adele: Ja I think it would be hard if you were constantly sick and constantly ill, missing work you know going to the doctor all the time. I would hate that.

Christina: when you are healthy with like your body and your mind and in everything… you go out and do things to work harder to go the extra mile…

Christina: …a lot of jobs are reliant on you being well and I think, so to provide for yourself and to provide for your family you actually need to be in optimal situation so you can retain your job. It sounds so depressing. But unfortunately, it’s the way that the world works now.

The WHO’s goal for, “All people in all countries [to] have at least such a level of health that they are capable of working productively and of participating actively in the social life in which they live” (World Health Organisation’s global strategy of Health for all by the year 2000, cited in Petersen & Lupton, 1996, p. 1) also illustrates this idea of ability of health to facilitate productivity, to “*work harder and go the extra mile”* and to avoid “*missing work”*. Harvey (2000) argues that within a capitalist society illness is defined as the “inability to go to work” (p. 106). In the above quotes we see how discourses around health take shape within a context of prominent neoliberal capitalist ideas and how these ideas permeate the way individuals talk about and experience health.

The discourses emphasising the importance of the freedom to work are also used when participants justify behaviours labelled “unhealthy”, by explaining that if they are not impairing their productivity then they are not really bad for one’s health. In addition, if these “unhealthy” behaviours enhance productivity they can be considered healthy for that situation.

Miranda: *I have one friend who, you know, she smokes weed everyday whenever she can but she is one of the most highly functional and productive people I’ve ever met!*…*she was just active all the time, and she says that she has got a lot of you know anxiety issues and if she doesn’t smoke weed um those you know those completely overwhelm her, and that’s why she smokes so much weed, it helps keep her calm and less anxious. Whereas if I were to smoke weed all day every day I would be the most lazy useless person, I would just sit in my room eating and watching series whereas she, that actually helps her handle the hectic pace at which she does things. So you know something like that- it’s hugely different according person to person um but also for some people helps them be more functional whereas with other people it makes them a lot less functional so…*

Miranda’s quote again emphasises the importance of individualised approaches to health that individuals are free to choose based on their specific circumstances. She also reinforces the idea that health is important in so far as it supports productivity. The friend that she describes was considered healthy despite the fact that she smoked cannabis (which she labels as an unhealthy practice) because she was able to overcome the limitations of her *“anxiety issues”* in order to make it possible for her to work and to work at a very fast pace. In this quote, Miranda defines health in relation to functionality, so if a behaviour improves how well one functions, in particular at work, then this could be reframed as a healthy behaviour.

#### Freedom, health and corporate wellness programs

The intersection of discourses of freedom, health and productivity is particularly interesting in the context of corporate wellness programs. Some corporations provide employees with a number of health resources and yearly check-ups involving lifestyle assessments in a bid to improve employee wellbeing. This pursuit is believed to improve productivity and efficiency. The idea is that if employees are healthy they will be happy, positive and productive (Cederström & Spicer, 2015; Davies, 2015). In Meredith’s quote below she demonstrates the increasingly invasive and regulatory nature of some corporate initiatives to promote wellness.

Meredith: I know at [company name] at the beginning of every- you tell your project leader this is what I’m doing here and these are my goals to exercise, it’s actually, the way they regiment it, it made me quite scared. You’ve gotta tell them that you wanna exercise this many times a week, you wanna do this this many times a week, and then at the end of the week you’ve gotta tick a form that says did you do this, did you do, this did you do that. So they can see if you’re keeping up with your own health goals which is cool, but like I said it regiments it, and it doesn’t become this free relationship and the enjoyment in exercise that it should be…Also eating healthily’s definitely on there, eat vegetarian once a week… your KPIs- key performance indicators, that’s what you sent in the beginning.

The distinction between having the freedom *to* work and being free *from* restrictive or imposing rules is complicated in this extract. Meredith describes how this company *“regiments”* an individual’s health behaviours, enforcing accountability to the company and imposing an obligation on employees to ensure that they are correctly maintaining their health. Although individuals appear to be free to decide what appropriate goals for them are, the kinds of activities which individuals need to commit to (exercising and eating healthy food) seem to be prescribed. The dynamic Meredith describes, where individuals propose certain goals and are then supposed to account for their performance at the end of each week, is an illustration of the productive nature of biopower (Foucault, 1977, 1978). The kind of power exerted over employees in this instance is inciting action.

The relationship between power and an understanding of freedom of choice is played out in the wellness program which intends to position a corporate demand for healthy workers as an opportunity for employees to select personal goals for themselves and regularly review their progress in order to optimise their health. The transference of responsibility from the company to the individual is seen in phrases like *“my goals”, “your own health goals”* and *“you wanna exercise this many times a week”*. In this way, the intention of the intervention is for individuals to internalise their own surveillance, adopting it and experiencing it as a free, personal choice for their own self-improvement. In this way the external disciplinary pressures are meant to successfully facilitate the disciplining of the self by the self (Foucault, 1977).

However, Meredith is critical of this corporate wellness strategy, and resists it by also constructing ideal health behaviours as existing outside of power relations between people. She says that an individual’s relationship to their own exercise should be *“free”* and that individuals should experience *“enjoyment”* from exercising which appears to be viewed as less likely when exercise is more obviously imposed on someone by an external agent. The company’s initiative to discipline employees is challenged because the use of power has been exposed. Meredith can see the company’s intentions to exploit her and as a result the illusion of free choices is disrupted. The way Meredith frames exercise outside of this context of more visible expressions of power as an act of free choice, illustrates how more subtle and pervasive disciplinary pressures are often invisible. The choices Meredith currently makes about exercising (she is not yet working at the company she describes above) are experienced as *“free”* from external impositions. This understanding and experience of individual freedom of choice functions to successfully conceal the other ways in which power acts to govern individual conduct.

Many companies have corporate wellness strategies that are less obviously regulatory than the one described by Meredith. They may offer team building exercises, healthy food options at work, and on-site exercise resources (Cederström & Spicer, 2015). These interventions are always positioned as aiming to facilitate individual employee choices to improve their own wellbeing. However, the goal of increased productivity underlies all corporate wellness interventions. If individuals become more productive, one would expect that they would have more time available as they would be able to complete their work more quickly and efficiently. Cederström and Spicer (2015) ask the question: “How should we use the time that has now been freed up? The answer it seems is to find new ways to be even more productive”. Poole (2012) argues that “the obsessive dream of productivity becomes a perfectly effective defense against its own realisation”. When there is no specific goal to reach, the pursuit of productivity, wellness or health becomes never-ending and we become trapped in the constant loop of trying to improve ourselves more and more. We become so concerned with this corporate sanctioned pursuit of a specific kind of perfection that we lose track of what we hoped to achieve as a result of our healthy lifestyle.

It is possible that individuals may be undecided about what they hope to achieve as a result of their improved health as, usually, the intention of these kinds of interventions (for them to work more and faster) is purposefully opaque. If health is viewed as key to performing any activity you could want, then it also means that improving health can be seen as mutually beneficial both to individuals as well as to corporations and *society* more generally. Corporate wellness programs are often viewed as a perk of working at a certain company as seen in the quote below, rather than as another imposition one’s job makes on one’s life.

Jo: But if businesses did encourage that like [name of business], they have their own gym on site they encourage people to use it. That’s really good. Quite a few businesses are taking that on now. Gyms are including lunch hours you know and classes where you don’t sweat as much so you can go back to work and not have to shower so there’s that, that’s good as well.

This idea of health enabling a freedom to work is facilitated by the value our societies place on working and earning money. Definitions of success often rest heavily on material wealth and having the capacity to purchase symbols of success or certain lifestyles that are seen as indicative of success and worth (James, 2007). The ideal of a social responsibility and the mandate to be productive citizens relates to the “duties” discourse mentioned by Petersen and Lupton (1996). The individual must fulfil her duties and obligations through hard work and dedication in order to achieve the status of successful citizen. It is interesting that within the discourse of freedom there is also evidence of this duties discourse, as the two seem in some ways contradictory. It is common though, for ideas of freedom to be presented alongside ideas of responsibility and so this construction of freedom which carries with it certain aspects of duty is facilitated and upheld within our cultural context. As Eleanor Roosevelt said: “with freedom comes responsibility”. While healthiness is associated with freedom, it is clear that within this corporate context, freedom is increasingly redefined as the freedom to be productive worker.

## Discussion

Rose (1998) argues that the ethic of the “free, autonomous self seems to trace out something quite fundamental in the ways in which modern men and women have come to understand, experience, and evaluate themselves, their actions, and their lives” (p. 2). This is seen above in the ways in which individuals give an account of their health and of themselves in relation to the notion of health. Health is valued as a tool to maximise freedom, and to construct a self that transcends the materiality of the body and the limitations that are associated with it. The neoliberal emphasis on productivity and individual responsibility facilitates this discourse and is supported by it. A self that is active, productive, valuable, hopeful and whole is constructed through the use of this discourse, and while individuals are enabled to view themselves as good people it also brings with it similar victim-blaming noted in other discourses of health: if an individual is suffering from some sort of health problem, all they need to do is choose to overcome the limits of their body by working on it and transforming it. And further, that here failing to overcome these limits through personal effort becomes not simply a practical failure, but a moral one. This discourse also contains an element of fantasy, the idea that anything is possible and that we can achieve anything we set our minds to. This hopefulness directed inwards places the responsibility for the social obligations of both healthiness and freedom itself on the individual. This reproduces the belief that individuals need not concern themselves with collective action, or transforming social structures or systems that place vulnerable people at risk. Instead, it advances the idea that transformation and improvement should be directed at, and by, the self.

## Conclusion

This paper explores how popular ideas of health are constructed using discourses of freedom, highlighting the potential implications these constructions have for both individuals’ personal experiences of health, and for broader social structures. The analysis outlines the complex ways in which healthiness was constructed as freeing, highlighting how these shape individual lifestyle choices. In addition, the analysis provides a critique of the influence of neoliberal capitalism in these freedom/health discourses, particularly in the context of corporate wellness programmes. It has been argued that constructions of health that draw on discourses of freedom serve individuals as they allow them to resist certain prescriptive health ideals and facilitate a personal sense of hope. At the same time, however, they function negatively by concealing the ways in which health discourses can be individualising, stigmatising, and contribute to an increasingly marginalising social system by detracting from the importance of collective social welfare resources.

Limitations and future research

One of the limitations of this study is related to the lack of diversity in the sample. Although individuals from a range of “racial” categories and class backgrounds were interviewed, the majority of the participants identified as “white” and were from middle class backgrounds. Therefore, whilst the analysis provides an in-depth exploration of a small group’s use of health discourses, discourses used by individuals from poorer or rural backgrounds or from racial categories other than “white” may not have come through as strongly as they would have had the sample been more diverse. The relatively small percentage of individuals who were from working-class backgrounds may be partially a result of the level of wealth or leisure time required to actively engage in the kinds of health promotion activities that are socially valued. A relatively good level of health- which is likely related in certain ways to class and race groups- may have played a role in the willingness of participants to be interviewed for this study.

The lack of diversity mentioned above also relates to the reported health statuses of the participants. The group of individuals who were interviewed all described themselves as relatively healthy at the time of the interview, none were chronically ill and none were disabled. This may have influenced the kinds of discourses which were taken up and the ways in which these discourses played a role in the constitution of subjects. This also meant that this study wasn’t able to explore the effects of these discourses on the subjectivities and experiences of those who would be more marginalised by them. This is an important area to explore in future studies.
